# ChAdOx1 nCoV-19 (AZD1222) vaccine-induced Fc receptor binding tracks with differential susceptibility to COVID-19

**DOI:** 10.1038/s41590-023-01513-1

**Published:** 2023-06-15

**Authors:** Paulina Kaplonek, Deniz Cizmeci, Gaurav Kwatra, Alane Izu, Jessica Shih-Lu Lee, Harry L. Bertera, Stephanie Fischinger, Colin Mann, Fatima Amanat, Wenjun Wang, Anthonet L. Koen, Lee Fairlie, Clare L. Cutland, Khatija Ahmed, Keertan Dheda, Shaun L. Barnabas, Qasim Ebrahim Bhorat, Carmen Briner, Florian Krammer, Erica Ollman Saphire, Sarah C. Gilbert, Teresa Lambe, Andrew J. Pollard, Marta Nunes, Manfred Wuhrer, Douglas A. Lauffenburger, Shabir A. Madhi, Galit Alter

**Affiliations:** 1 Ragon Institute of MGH, MIT, and Harvard, Cambridge, MA USA; 2 South African Medical Research Council Vaccines and Infectious Diseases Analytics Research Unit, Faculty of Health Sciences, University of the Witwatersrand, Johannesburg, South Africa; 3 Department of Science and Innovation/National Research Foundation South African Research Chair Initiative in Vaccine Preventable Diseases Unit, University of the Witwatersrand, Johannesburg, South Africa; 4 African Leadership in Vaccinology Expertise, Faculty of Health Sciences, University of the Witwatersrand, Johannesburg, South Africa; 5 Center for Infectious Disease and Vaccine Discovery, La Jolla Institute for Immunology, La Jolla, CA USA; 6 Department of Microbiology, Icahn School of Medicine at Mount Sinai, New York, NY USA; 7 Center for Proteomics and Metabolomics, Leiden University Medical Center, Leiden, the Netherlands; 8 Wits Reproductive Health and HIV Institute, Faculty of Health Sciences, University of the Witwatersrand, Johannesburg, South Africa; 9 Setshaba Research Centre, Tshwane, South Africa; 10 Division of Pulmonology, Groote Schuur Hospital and the University of Cape Town, Cape Town, South Africa; 11 Faculty of Infectious and Tropical Diseases, Department of Immunology and Infection, London School of Hygiene and Tropical Medicine, London, UK; 12 Family Centre for Research With Ubuntu, Department of Paediatrics, University of Stellenbosch, Cape Town, South Africa; 13 Soweto Clinical Trials Centre, Soweto, South Africa; 14 Perinatal HIV Research Unit, Faculty of Health Sciences, University of the Witwatersrand, Johannesburg, South Africa; 15 Jenner Institute, Nuffield Department of Medicine, University of Oxford, Oxford, UK; 16 Oxford Vaccine Group, Department of Paediatrics, University of Oxford, and the NIHR Oxford Biomedical Research Centre, Oxford, UK; 17 Department of Biological Engineering, Massachusetts Institute of Technology, Cambridge, MA USA

**Keywords:** DNA vaccines, Viral infection

## Abstract

Despite the success of COVID-19 vaccines, severe acute respiratory syndrome coronavirus 2 (SARS-CoV-2) variants of concern have emerged that can cause breakthrough infections. Although protection against severe disease has been largely preserved, the immunological mediators of protection in humans remain undefined. We performed a substudy on the ChAdOx1 nCoV-19 (AZD1222) vaccinees enrolled in a South African clinical trial. At peak immunogenicity, before infection, no differences were observed in immunoglobulin (Ig)G1-binding antibody titers; however, the vaccine induced different Fc-receptor-binding antibodies across groups. Vaccinees who resisted COVID-19 exclusively mounted FcγR3B-binding antibodies. In contrast, enhanced IgA and IgG3, linked to enriched FcγR2B binding, was observed in individuals who experienced breakthrough. Antibodies unable to bind to FcγR3B led to immune complex clearance and resulted in inflammatory cascades. Differential antibody binding to FcγR3B was linked to Fc-glycosylation differences in SARS-CoV-2-specific antibodies. These data potentially point to specific FcγR3B-mediated antibody functional profiles as critical markers of immunity against COVID-19.

## Abstract (web summary)

Kaplonek et al. provide a prospective look at ChAdOx1 nCoV-19-vaccinated individuals who go on to experience breakthrough COVID infections. They report an antibody Fc profile characterized by higher FcgR2b and reduced Fcgr3b for individuals who are more susceptible to breakthrough infections.

## Main

The development of several SARS-CoV-2 vaccines has substantially helped reduce morbidity and mortality worldwide. However, breakthrough infections among fully vaccinated people by variants of concern (VOCs) have risen globally^1–6^. Despite the increase in breakthrough cases, vaccine-mediated protection against severe disease and death remains stable in the setting of detectable antibodies^6–8^. Breakthrough infections and COVID-19 are on the rise due to waning immunity^9,10^ and mutational escape from neutralizing antibodies induced by the ancestral vaccine strain^11,12^. However, although neutralizing antibodies have been associated with protection against infection across several vaccine trials^6,13,14^, simple binding titers also appear to track robustly with protective immunity^15–17^. In addition, emerging analyses in animal models suggest that antibody Fc-effector functions, including opsonophagocytosis, correlate with protective immunity after natural infection^18,19^, convalescent plasma therapy^20–22^ and monoclonal therapy^23,24^. Yet, whether particular Fc profiles are linked to vaccine-mediated protection in humans remains undefined but could provide critical insights to guide boosting strategies and inform next-generation, variant-specific vaccine design.

The Oxford–AstraZeneca ChAdOx1 nCoV-19 (AZD1222) vaccine, one of the earliest vaccine platforms, demonstrated 66.7% (95% confidence interval 57.4–74.0) efficacy against symptomatic ancestral SARS-CoV-2 infection^25^. Despite the induction of robust vaccine-specific immunity, mutations arising in the spike protein in diverse regions of the world, in the face of waning immunity, have resulted in increasing breakthrough infections^26^. Nevertheless, rates of severe disease and death have not increased proportionally compared with breakthrough infections and mild COVID-19, arguing that vaccine-induced immunity continues to provide robust protection against severe illness through alternative mechanisms^27–29^.

Beyond neutralization, antibodies leverage diverse antiviral functions through their ability to recruit innate immune effector functions via the antibody constant domain (Fc domain) interactions with Fc receptors (FcRs) found on all innate immune cells^30^. FcRs are expressed in different combinations on different cell types, enabling antibodies to drive disparate functions^31,32^. Eight canonical FcRs have been described in humans^33^, which include FcRs for all immunoglobulins IgM, IgA, IgE and IgG. For IgG, five subtypes have been described, including one high-affinity receptor, the FcγR1, and four low-affinity IgG-binding FcRs, which largely tune IgG-mediated Fc-effector function, including the activating FcγR2a, the inhibitory FcγR2b, the activating FcγR3a and the glycosylphosphatidylinositol (GPI)-anchored FcγR3b receptors^32^. Although FcγR2a, FcγR2b and FcγR3a are expressed on several cell types, FcγR3B is exclusively expressed on neutrophils^34,35^. Fc-mediated effector functions have been linked to protection against several infectious diseases, including influenza^36^, malaria^37^, human immunodeficiency virus (HIV)^38^, tuberculosis^39^, Ebola virus^40^ and COVID-19 in hamsters and nonhuman primates (NHPs)^18,41^. Likewise, the ChAdOx1 nCov-19 vaccine was shown to induce SARS-CoV-2-specific antibodies^14^ that could facilitate Fc-effector functions across VOCs^42,43^. Yet, although antibody titers and neutralization were associated with protective immunity against the first wave of the original variant of SARS-CoV-2 (refs. ^44,45^), vaccine correlates of immunity against emerging VOCs that evade neutralization have yet to be precisely defined.

To define whether additional antibody functions track with differential protection against COVID-19, we deeply profiled the humoral immune response in individuals enrolled in the ChAdOx1 nCoV-19 vaccine clinical trial in South Africa performed between June and November 2020 (COV005), in which 92.9% of primary endpoint cases were caused by the beta (B.1.351) SARS-CoV-2 VOC, all of which were mild to moderate in severity^26^. SARS-CoV-2-specific antibody titers and their FcR-binding profiles were examined across the WT and beta (B.1.351) VOC spike and receptor-binding domain (RBD) variant antigens at peak immunogenicity (at least 2 weeks after the final immunization) across vaccinees who developed beta-variant-induced COVID-19 (*n* = 30), as well as a demographically matched set of vaccinated controls who remained free of COVID-19 (*n* = 140) (ref. ^26^). Although limited differences in wild-type (WT) and beta-specific binding antibody titers were observed across vaccinees, substantial differences were noted in isotype and FcR-binding profiles across both WT and beta-specific humoral immune responses, linked to distinct inflammatory properties. These data suggest that divergent FcR-binding profiles, with specific capabilities of arming inflammatory cascades, represent new biomarkers that may contribute to immunity against VOC-induced COVID-19.

## Results

### Characteristics of vaccinated participants

To evaluate humoral correlates of immunity against COVID-19, beyond neutralization, in the present study we exploited a systems serology approach to deeply profile the humoral immune response to the WT and beta VOCs of SARS-CoV-2 in a case–control substudy of the ChAdOx1 nCoV-19 vaccine clinical trial in South Africa, performed between June and November 2020 (COV005). Volunteers aged 18–62 years (median age 31 years) were immunized with two doses of ChAdOX1 nCoV-19 administered 4 weeks apart. Participants were self-monitored for COVID-19 and nasal swabs were collected for nucleic acid amplification testing in individuals who experienced COVID-19 symptoms^46^. The SARS-CoV-2 infections were virologically confirmed, defined with a nucleic acid amplification test-positive swab, and only COVID-19-reported cases with confirmed beta VOC breakthrough >14 days post-boost were included in this analysis (*n* = 30). Fully vaccinated, demographically matched individuals without any SARS-CoV-2 infection were used as controls (*n* = 140) for this analysis (Table 1), matched based on sex, age, body mass index (BMI) and race. Volunteers who were anti-nucleoprotein IgG seropositive at first vaccination were excluded from the study. Additional demographic factors were not identified as disease modifiers in the original clinical trial^47^. Given the goal of identifying correlates of modified COVID-19 disease, rather than transmission, asymptomatic seroconverters were not included in this analysis. Vaccine-induced humoral profiles across the groups were all profiled 2 weeks post-boost. Systems serology was applied to all samples in a blinded fashion, capturing WT SARS-CoV-2 RBD-, N-terminal domain (NTD)-, spike (S)-, S1- and S2- and beta variant S- and RBD-specific antibodies (IgG1, IgG3, IgM and IgA), and Fcγ-receptor-binding profiles (Fcγ2A, Fcγ2B, Fcγ3A and Fcγ3B).

**Table 1:** Characteristics of vaccinated participants

	Overall^a^	COVID-19^+^		COVID-19^−^
**Enrolled,** ***n***	170	30		140
**Male,** ***n*** **(%)**	108 (63.5)	16 (55.3)		92 (65.3)
**Median age, years (IQR)**	31 (18–62)	31 (19–58)		31 (18–62)
**18 to <45,** ***n*** **(%)**	137 (80.5)	25 (83)		112 (80)
**45 to <60,** ***n*** **%**	32 (18.8)	5 (17)		27 (19.2)
**>60,** ***n*** **(%)**	1 (0.5)	0 (0)		1 (0.7)
**BMI,** ***n*** **(%)**				
**Underweight**	21 (12.3)		4 (13.3)	17 (12.1)
**Normal**	89 (52.3)		16 (53.3)	73 (52.1)
**Overweight**	38 (2,239)		10 (33.4)	28 (20)
**Obese**	22 (12.9)		0 (0)	22 (15.7)
**Health-care workers**	15 (8)		3 (7.9)	12 (8)
**Race,** ***n*** **(%)**				
**Black**	134 (78.8)		27 (90)	107 (76.4)
**White**	22 (12.9)		3 (10)	19 (13.5)
**Mixed**	11 (6.4)		0 (0)	11 (7.8)
**Other**	3 (1.6)		0 (0)	3 (2.1)
**COVID-19 comorbidities,** ***n*** **(%)**				
**Hypertension**	4 (2.1)		0 (2.6)	3 (2.1)
**Respiratory system disorders**	8 (4.3)		0 (2.6)	8 (5.7)
**Diabetes**	0 (0)		0 (2.6)	0 (0)
**Tobacco use**	72 (42.3)		7 (23.3)	65 (46.4)
**Alcohol use**	84 (49.4)		16 (53.3)	68 (48.5)

^a^Overall includes all COVID-19^+^ and COVID-19^−^ participants’ demographic data.

IQR, interquartile range.

### Vaccinees who developed COVID-19 exhibit functionally divergent antibody profiles

**Fig. 1:**
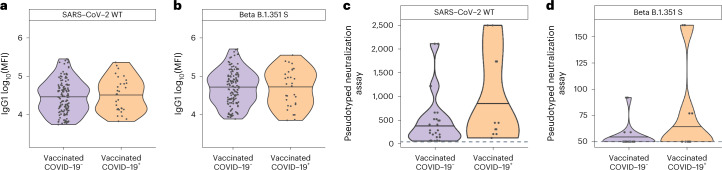
Equivalent WT and beta (B.1.351) IgG1 S-specific antibody levels and neutralization titers across vaccinees who either developed COVID-19 or were uninfected. **a**,**b**, Violin plots showing the univariate comparison of WT (**a**) and beta B.1.351 (**b**) SARS-CoV-2 S-specific IgG levels between vaccinees who resisted COVID-19 (*n* = 140) and individuals who developed COVID-19 (*n* = 30) over the study period. MFI, mean fluorescence intensity. **c**,**d**, SARS-CoV-2 WT (**c**) and beta B.1.351 (**d**) neutralization titers measured for vaccinees who resisted COVID-19 (*n* = 28) or developed beta VOC COVID-19 (*n* = 12). A Mann–Whitney *U*-test was used to define differences and the Benjamini–Hochberg method was used to adjust for multiple comparisons, with an adjusted *P* (*P*_adj_): ^***^P < 0.001; ^**^P< 0.01; ^*^P < 0.05. Source data

Although previous studies have found an association between antibody-binding titers and neutralization with protection across phase 3 COVID-19 vaccine trials^15,48^, WT S IgG titers and neutralization were not different across vaccinees who did or did not develop beta-variant COVID-19 after ChAdOX1 nCoV-19 immunization (Fig. 1a–d). These data suggest that antibody binding alone cannot explain vulnerabilities in the vaccine-induced humoral immune response against the beta VOC.

**Fig. 2:**
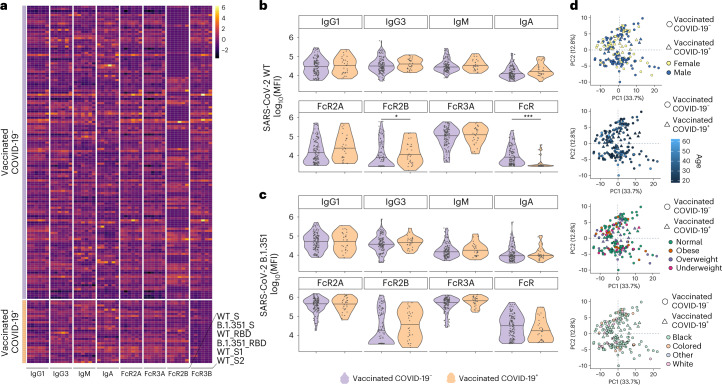
Diverging antibody Fc profiles across ChAdOx1 nCoV-19 vaccinees who did or did not develop COVID-19. **a**, Heatmap summarizing the SARS-COV-2 WT and beta (B.1.351)-specific IgG1, IgG3, IgA1 and IgM titers, as well as the ability of SARS-CoV-2-specific antibodies to bind to the low-affinity Fcγ-receptors (FcγR2A, Fcγ2B, Fcγ3a and Fcγ3b) across the vaccinees who did not (*n* = 140) or did develop COVID-19 (*n* = 30). Each column represents a distinct feature that was analyzed in the plasma and each row a different plasma sample. Titers and FcR data were first log(transformed) and *z*-scores are shown for comparison. **b**,**c**, Violin plot showing univariate comparisons of WT (**b**) and beta (**c**) SARS-CoV-2 S-specific Fc-antibody profiles between the groups. A Mann–Whitney *U*-test, with a correction for multiple comparison using the Benjamini–Hochberg method, was used to test for differences across the groups. **d**, A PCA applied to all samples and data, including vaccinees who did and did not develop COVID-19, to examine the impact of different demographic parameters on antibody profiles. In each panel, samples are colored based on sex, age, BMI and race, demonstrating limited effects of these demographic characteristics on shaping vaccine-induced humoral profiles. ^***^P < 0.001; ^**^P < 0.01; ^*^P < 0.05. Source data

To better understand other potential differences in the vaccine-induced humoral immune response that may explain differences in protection against COVID-19 in the setting of beta VOC infection, we next compared the overall humoral immune response to the vaccine (WT) and the beta VOC S antigens^26^. SARS-CoV-2-specific humoral responses were detected across all vaccinees, marked by the selection of several antibody isotypes and subclasses, with the capability of binding to several IgG FcRs (Fig. 2a and Supplementary Fig. 1). Although each individual exhibited a unique overall isotype/subclass/FcR-binding profile, some distinct differences in Fc profiles were noticeable across the cases and controls, including reduced SARS-CoV-2-specific antibody FcγR2B binding in blocks of vaccinees who did not develop COVID-19 and a uniformly lower-level, SARS-CoV-2-specific antibody FcγR3B binding in vaccinees who later developed COVID-19. The univariate analysis confirmed no differences in SARS-CoV-2-specific IgG1, IgG3 and IgM titers to the WT- and beta-S between beta VOC breakthrough cases and individuals who did not develop COVID-19 (Fig. 2b,c). Both WT- and beta-S-specific, FcγR2B-binding levels were higher in vaccinees who developed COVID-19, after correction for multiple comparisons. Conversely, WT S-specific FcγR3B-binding levels were significantly lower in individuals who ultimately developed COVID-19 (Fig. 2b,c). Similar Fc-profile differences were observed for RBD-specific responses (Supplementary Fig. 2). Importantly, principal component analysis (PCA) demonstrated no variation in vaccine-induced Fc profiles based on sex, age, BMI and race (Fig. 2d and Supplementary Fig. 3). These data point to qualitative differences at peak immunogenicity in isotype and FcR-binding profiles across vaccinees who developed COVID-19 after a primary beta VOC infection.

### FcγR3B-binding profiles discriminate vaccinated individuals who resist or develop COVID-19

**Fig. 3:**
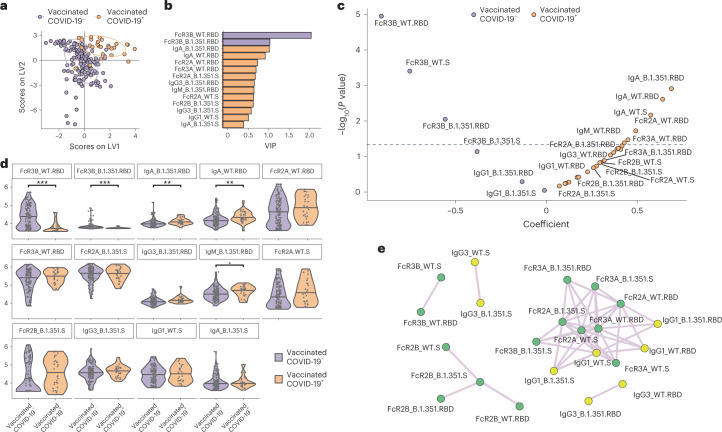
FcγR3B-biased SARS-CoV-2-binding responses track with enhanced protection from beta VOC-induced disease. **a**, A LASSO used to reduce the feature dimensionality and ultimately select antibody features that discriminated between vaccinees who resisted COVID-19 and those who developed the disease over the study period. The PLS-DA was then used to visualize the separation between the samples based on the LASSO-selected features, where each dot represents an individual vaccinee. Violet dots represent vaccinees who resisted COVID-19 disease over the study period and orange dots the vaccinees who developed COVID-19 over the study period. **b**, Bar graph showing the ranking of the LASSO-selected features based on a VIP score. **c**, The LME model depicting the overall differences in antibody features between individuals who resisted COVID-19 (left side) and individuals who developed COVID-19 (right side). The models were corrected for sex, age, BMI, race, alcohol and smoking status. The *x* axis depicts the effect size between the groups and the *y* axis shows the statistical significance. The hatched line depicts the significance cut-off after multiple comparisons. **d**, Violin plots showing the univariate comparisons of LASSO-selected features between the vaccinees who resisted COVID-19 and those who developed disease over the study period. Statistical differences were defined using a Mann–Whitney *U*-test and a correction for multiple comparisons, using the Benjamini–Hochberg method, and all *P* values were adjusted (^***^P < 0.001; ^**^P < 0.01; ^*^P < 0.05). **e**, Network analysis showing the additional antibody features, which were correlated with the LASSO-selected features and are likely to be important in driving the separation. The network was built using a threshold of absolute Spearman’s ρ < 0.7 and Benjamini–Hochberg-adjusted *P*_adj_ < 0.01. Nodes were colored based on the type of measurement: antibody titers and FcR binding. The connecting lines denote all positive correlations (no negative correlations were observed). Source data

Given the highly coordinated nature of isotype/subclass and FcR-binding profiles, we next aimed to conservatively define the minimal vaccine-induced Fc differences that most effectively distinguished vaccinees who ultimately developed COVID-19 compared with vaccinees who did not develop beta VOC COVID-19. Both WT and beta VOC-specific humoral data were integrated and a least absolute shrinkage and selection operator (LASSO) was first applied to conservatively reduce the overall features to the minimal number of vaccine measurements that could discriminate between the two groups. The data were then visualized using a partial least squares discriminant analysis (PLS-DA). Almost-complete separation was noted in vaccine profiles across vaccinees who ultimately developed COVID-19 compared with those who remained free of COVID-19 for the duration of the study (Fig. 3a). Two features were exclusively enriched among vaccinees who did not develop COVID-19, including both WT and beta VOC RBD-specific, FcγR3B-binding antibody levels (Fig. 3b). Moreover, using an orthogonal approach, a mixed linear-effects (MLE) model, adjusted for all potential demographic confounders (such as age, sex, BMI, race, health status, smoking and drinking), identified similar diverging humoral immune features enriched among vaccinees who resisted COVID-19 compared with those who developed COVID-19. Specifically, higher levels of multiple FcγR3B-binding antibody features were selectively enriched among vaccinees who did not develop COVID-19. In contrast, higher levels of IgA and FcγR2A-binding antibodies were enriched among vaccinees who experienced beta VOC breakthrough COVID-19 (Fig. 3c). Similarly, across the LASSO/PLS-DA, 11 antibody features were selectively enriched among vaccinees who developed COVID-19, including both WT and beta RBD-specific IgA, WT or beta-RBD-specific FcγR2A, IgG3, IgM, FcγR2B, IgG1 and IgA to WT or beta VOC S-specific binding antibodies (Fig. 3d). Moreover, a cocorrelate analysis was further performed using the LASSO-selected features to fully dissect the relationship between the differentially enriched biomarkers. Importantly, all FcγR3B features were linked to each other (Fig. 3e), suggesting that vaccinees who did not develop COVID-19 for the duration of the study elicited a highly coordinated broad FcγR3B-binding response across both RBD and S. Conversely, three separate networks emerged linked to the markers enriched in vaccinees who developed COVID-19, including small networks of FcγR2B WT S, WT RBD and beta VOC RBD features, and a more extensive network of IgG1, FcR2A, FcγR3A WT and beta VOC features. Thus, despite equivalent S-specific antibody titers induced after vaccination across vaccinees who did or did not resist COVID-19, these data demonstrated that Fc profiles were highly divergent across the groups, marked by qualitatively distinct capabilities of interacting with FcRs.

**Fig. 4:**
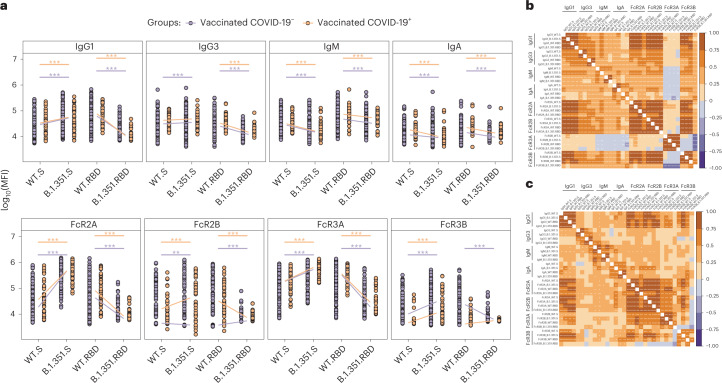
WT and beta RBD and S-specific Fcγ2B and Fcγ3B differ across vaccinees who developed COVID-19 or resisted disease. **a**, Dot plots showing the univariate comparisons of S- and RBD-specific antibody levels to the WT or beta S (left) or RBD (left) across the vaccinees who resisted or developed COVID-19 over the study period. Differences were defined using a Wilcoxon’s rank-sum test, and all *P* values were corrected for multiple comparisons using the Benjamini–Hochberg method with: ^***^P < 0.001; ^**^P < 0.01; ^*^P < 0.05. **b**,**c**, Correlation matrices depicting the Spearman’s correlations between SARS-CoV-2 WT and beta B.1.351 S-specific antibody features in vaccinated individuals who resisted (**b**) or developed (**c**) COVID-19. Significance values were corrected for multiple comparisons using the Benjamini–Hochberg method and shown as: ^***^P < 0.001; ^**^P < 0.01; ^*^P < 0.05. The lower triangle shows *P* values, whereas the upper triangle shows *P*_adj_ values. Source data

Given the presence of neutralizing antibody-escape mutations in the RBD of the beta VOC^49^, we next compared differences in S- and RBD-specific Fc-binding profiles across WT and beta VOC-S or -RBD antigens. It is interesting that S-specific IgG1, IgG3, FcγR2A and FcγR3A responses were enhanced to the beta VOC for both cases and controls (Fig. 4a). Conversely, largely reduced S-targeting IgM and IgA levels were observed for the beta VOC compared with the WT across the groups. Similarly, IgG1, IgG3, IgM, IgA, FcγR2A and FcγR3A were lower to the beta RBD compared with the WT across both cases and controls. However, as observed in the univariate and multivariate analyses, SARS-CoV-2-specific FcγR2B and FcγR3B binding diverged across the two groups, marked by different directions of S- and RBD-specific responses across the WT and beta VOC. Specifically, beta S-specific FcγR2B binding was higher than to WT in individuals who ultimately developed COVID-19 but remained low in uninfected vaccinees. Moreover, FcγR2B binding was detectable to the WT RBD in both groups, but not to the beta VOC RBD, arguing for a limited role of FcγR2B-recruiting, RBD-specific antibodies in the immunity against beta VOC infection and disease. In addition, FcγR3B binding to both the WT and the beta VOC S and RBD were globally lower in vaccinees who ultimately developed COVID-19 compared with those who did not, pointing to a critical role for both S- and RBD-specific FcγR3B-binding antibodies in beta VOC immunity (Fig. 4a). Moreover, correlational analysis of the WT- and VOC-induced humoral immune responses across vaccinees who did not develop COVID-19 pointed to a negative correlation between FcγR2B-binding antibodies and IgA and IgM responses (Fig. 4b), but more diffuse coordination of all FcR-binding profiles with isotype selection in individuals who were ultimately infected (Fig. 4c). Robust relationships, albeit not perfect, were observable between IgG titers and each FcR except for FcγR3B binding for individuals who developed COVID-19 (Supplementary Fig. 4). The fact that IgG titers were equivalent across individuals who ultimately developed COVID-19 and ones who remained uninfected, but had FcγR3B binding that was significantly lower, further emphasizes the disconnection between titers and particular FcγR-binding responses. Thus collectively, Fc-profile differences, specifically S-specific FcγR2B and S- and RBD-specific FcγR3B binding after vaccination, may represent biomarkers of protective humoral immunity against beta VOC-induced COVID-19 after ChAdOx1 nCoV-19 vaccination.

### Antibody effector functions and cytokine production depends on FcγR2B/3B-binding profile

**Fig. 5:**
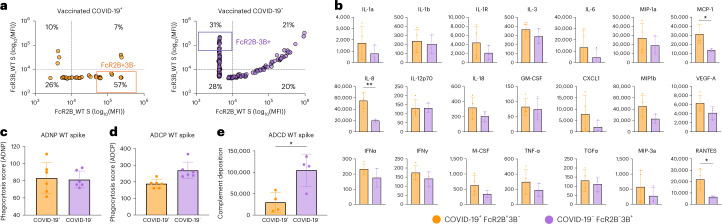
FcγR2B^+^3B^−^- and FcγR2B^−^3B^+^-binding IgG show different abilities to drive antibody-dependent effector functions as well as cytokine and chemokine production. **a**, The selection strategy of COVID-19^+^FcR2B^+^3B^−^ and COVID-19^−^FcR2B^−^3B^+^ pools based on the ability of WT S-specific IgG to bind to FcγR2B and FcγR3B receptors. Subjects in the top quartile (marked with boxes) were selected and pooled (pool of *n* = 5). **b**, ADCD. **c**,**d**, ADCP (**c**) and ADNP (**d**) in COVID-19^+^FcR2B^+^3B^−^ and COVID-19^−^FcR2B^−^3B^+^ pools (pool of *n* = 5). Bars show the mean value with s.d. Dots represents replicates (*n* = 4 and *n* = 6). Samples were run in technical duplicates and two (ADCD) to three (ADCP and ADNP) biological replicates. Unpaired Student’s *t*-test and *P*_adj_ (^***^P < 0.001; ^**^P < 0.01; ^*^P < 0.05) were used. **e**, Cytokine production by isolated human neutrophils stimulated with COVID-19^+^FcR2B^+^3B^−^ and COVID-19^−^FcR2B^−^3B^+^ pools (pool of *n* = 5). Bars show the mean with s.d. Dots represent replicates (*n* = 4, technical duplicates of two biological replicates with different blood donors). Unpaired Student’s *t*-test and *P*_adj_ (^***^P < 0.001; ^**^P < 0.01; ^*^P < 0.05) were used. GM-CSF, granulocyte–macrophage colony-stimulating factor; IFNγ, interferon-γ; MCP, monocyte chemoattractant protein; MIP, macrophage inflammatory protein; TGFα; transforming growth factor α; TNF-α, tumor necrosis factor α; VEGF, vascular endothelial growth factor. Source data

Given the substantial differences in FcγR-binding properties across vaccinees who did or did not ultimately develop COVID-19, specifically related to differential FcγR3B- and FcγR2B-binding profiles, we generated plasma pools of equal numbers of vaccine samples that displayed discrete binding profiles to FcγR3B or FcγR2B (FcγR2B^+^FcγR3B^−^ 57% versus 20% and FcγR2B^−^FcγR3B^+^ 10% versus 31%, for vaccinated COVID-19^+^ and vaccinated COVID-19^−^, respectively), for deeper antibody Fc-functional characterization (Fig. 5a). Two pools were formed including: (1) plasma samples that displayed the highest binding to FcγR2B (mean fluorescent intensity (MFI) > 10^4^) but not FcγR3B (MFI < 10^4^), found largely in vaccinees who ultimately developed COVID-19 (COVID-19^+^FcR2B^+^3B^−^) (pool of *n* = 5) and (2) plasma samples with the most robust binding to FcγR3B (MFI > 10^4^) and lacking binding to FcγR2B (MFI < 10^4^), vastly enriched in vaccinees who resisted COVID-19 for the study period (COVID-19^−^FcR2B^−^3B^+^) (pool of *n* = 5) (Fig. 5a). Both pools mediated robust and equivalent antibody-mediated, S-specific neutrophil phagocytosis (Fig. 5b). Conversely, the FcγR3B^+^/FcγR2B^−^ plasma pool demonstrated a trend toward elevated monocyte phagocytosis (antibody-dependent cellular phagocytosis (ADCP)) and significantly higher complement fixing activity (antibody-dependent complement deposition (ADCD)) (Fig. 5c,d), pointing to conserved neutrophil uptake but functional differences across additional Fc-effector functions. However, as FcγR3B is almost exclusively expressed on neutrophils^32,34,50–52^, we further defined whether equivalent antibody-dependent neutrophil phagocytosis (ADNP) activity was accompanied by differences in neutrophil activation, using a whole-blood assay that also includes FcγR2B-expression cells (dendritic cells and B cells^53–55^) that may influence neutrophil activation and function. Cytokine and chemokine release profiles were compared across the FcγR2B^+^3B^−^ or FcγR2B^−^3B^+^ pools after neutrophil uptake of opsonized S-coated beads (Fig. 5e). Trends toward higher cytokine/chemokine release were observed in the presence of the COVID-19^+^FcγR2B^+^3B^−^ pool of opsonized beads compared with the COVID-19^−^FcγR2B^−^3B^+^ plasma pool (Fig. 5e). Specifically, significantly higher proinflammatory interleukin (IL)-8, chemoattractant protein 1 (MCP-1) responsible for neutrophil recruitment to the lungs, as well as RANTES—critical for homing and migration of effector T cells (CCL5)—were observed in the setting of COVID-19^+^FcγR2B^+^3B^−^ plasma profiles compared with profiles enriched in the controls^56^. These data point to equivalent neutrophil-mediated phagocytic clearance of immune complexes across FcγR2B^+^3B^−^ and FcγR2B^−^3B^+^ groups, probably due to equivalent FcγR2A binding, but striking differences in inflammatory responses after uptake, resulting in elevated cytokines and chemokines in the absence of FcγR3B, may contribute to inflammatory cascades, cellular infiltration and activation of immunity in the lung that may lead to COVID-19.

### Differential FcγR2B/3B binding and functions are linked to divergent Fc-fragment glycosylation patterns

**Fig. 6:**
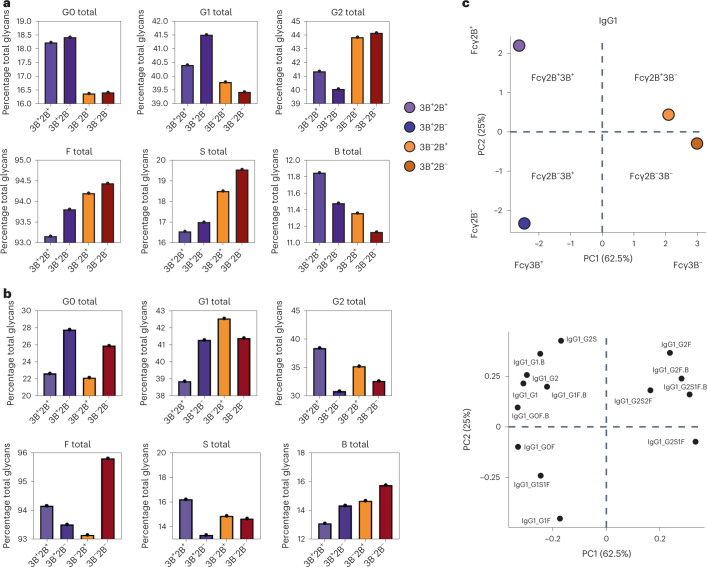
The capacity of SARS-CoV-2 S-specific IgG1 to bind to Fcγ2B and Fcγ3B receptors is regulated by the Fc-fragment glycosylation pattern. **a**,**b**, The overall representation (the percentage of total glycans) of major sugar classes, including galactose (agalactose: G0; single galactose: G1; digalactose: G2; fucose (F), sialic acid (S) and a bisecting GlcNAc (B)) across SARS-CoV-2 S-specific low and high FcγR2- and FcγR3B-binding IgG1 (FcγR2B^+^3B^+^, FcγR2B^+^3B, FcγR2B^−^3B) (**a**) and total antigen unspecific^+^, FcγR2B^−^3B^−^ groups (**b**) measured by LC–MS. Each bar shows the average of two replicates for FcγR2B^+^3B^+^, FcγR2B^+^3B^−^, FcγR2B^−^3B^+^ and FcγR2B^−^3B samples (pool of *n* = 5 per sample). **c**, PCA applied to all samples and data to examine the impact of various glycoforms on FcγR2B and FcγR3B binding for IgG1. Source data

IgG binding to FcRs is regulated by differences in Fc-subclass selection and Fc glycosylation^32,57^. Whereas IgG3 demonstrated a tendency toward higher levels in cases, we sought to determine whether Fc-glycosylation changes on the dominant circulating IgG, IgG1, could explain differences of binding across FcRs. Thus, we generated plasma pools with FcγR2B^+^3B^−^-, FcγR2B^−^3B^−^-, FcγR2B^+^3B^+^- and FcγR2B^−^3B^+^-binding profiles (all pools of *n* = *5*). Specifically, plasma samples that fell into one of these SARS-CoV-2 S-specific, antibody-binding profiles were pooled and S-specific antibodies were purified before masss spectrometry (MS) analysis of tryptic glycopeptides, and glycosylation on IgG1 Fc glycopeptides was analyzed. Four sugars are added in variable amounts to form the IgG Fc glycan, including galactose (agalactose: G0; single galactose: G1; digalactose: G2), fucose (F), sialic acid (single: S1; disialylated: S2) or a bisecting *N*-acetylglucosamine (GlcNAc; B) (refs. ^58,59^). A comparison of the overall representation of these major sugar classes across the plasma pools revealed differences across FcγR3B^+^- and FcγR3B^−^-binding antibodies (Fig. 6a). Specifically, FcγR3B^+^-binding antibodies harbored less galactose, less fucose, less sialic acid and more bisecting GlcNAc. In contrast, bulk antibody glycan profiles were distinct from S-specific antibody profiles, pointing to antigen-specific glycan differences and the importance of vaccine-specific glycosylation that may account for differential FcR binding and antibody functional activity (Fig. 6b).

Yet, given the potential for unique combinations of the carbohydrates that ultimately lead to the formation of the full N-linked glycan, we finally aimed to build a multivariate profile using Fc-glycan frequencies to capture the specific Fc glycans that were differentially enriched across antibodies able to engage FcγR3B and/or FcγR2B (Fig. 6c). Using an unsupervised PCA, performed on all IgG1 glycopeptide Fc-glycan structures, diverging Fc-glycan profiles were observed across all four FcR-binding plasma pools. Specifically, principal component 1 (PC1) accounted for 62.5% of the variance in the glycan samples, splitting Fc profiles according to the ability of antibodies to engage FcγR3B binding (left) or not (right). Along the PC1 axis, an enrichment of glycopeptides without galactose, sialic acid or fucose was observed on IgG1 that bound preferentially to FcγR3B. Conversely, PC2 accounted for 25% of the Fc-glycopeptide variation across the samples, pointing to glycan profiles with higher levels of bisection of IgG1 that bound preferentially to FcγR2B (top). Thus, differences in vaccine-induced Fc glycosylation, particularly lower galactosylation and fucosylation, may play a role in shaping more functional antibodies able to recruit FcγR3B^+^ functions that may be key to limiting inflammatory activation of neutrophils on opsonophagocytic uptake of the virus.

## Discussion

Despite the robust success of the ChAdOx1 nCoV-19 vaccine trial in the UK and Brazil against the WT and alpha variant of SARS-CoV-2 (ref. ^60^), protection against mild-to-moderate disease due to the beta VOC was not observed in South Africa, where most of the primary endpoint cases were due to the beta variant of SARS-CoV-2 (ref. ^26^). However, limited to no cases of severe disease or death were noted in the trial, pointing to persistent vaccine-mediated protection against the most vital endpoints, in the absence of transmission blockade^27–29,61,62^. With the global rise of VOCs that have begun to progressively break through vaccine-induced immunity more effectively, a more profound understanding of mediators of immunity, in addition to neutralization, is urgently needed. Moreover, NHP studies have pointed to complementary roles for both cellular and non-neutralizing functional humoral immunity in persistent protection against VOCs. For example, although T cell depletion was associated with a loss of viral control in only the upper respiratory tract and not the lung of NHPs, the transfer of subneutralizing, polyclonal, highly functional antibodies led to control of viral replication and accelerated clearance of the virus across both the upper and the lower respiratory tracts^63,64^. Similarly, in the present study, we found that, although neutralization and binding titers at peak immunogenicity were unable to predict protection against beta VOC COVID-19 breakthroughs, the presence of ChAdOx1 nCoV-19 vaccine-induced FcR-binding antibodies strongly diverged across vaccinees who ultimately developed COVID-19 compared with those who did not, pointing to unexpected humoral biomarkers of protection against beta-VOC-driven COVID-19.

Antibody effector functions have been implicated in protection against a wide array of pathogens^36–40,65^. In the present study, despite the presence of equivalent vaccine-induced, antibody-binding titers, we observed divergent vaccine-induced, FcR-binding antibody profiles, marked by enhanced and preferential FcγR3B binding in individuals who did not develop COVID-19, compared with elevated inflammatory isotypes/subclass/FcR antibody-binding profiles (IgA and IgG3) in vaccinees who ultimately developed COVID-19. These data suggest that the induction of highly functional isotype/subclass/FcR-binding profiles may not be sufficient to prevent COVID-19, in the absence of FcγR3B binding. A previous work highlighted the preferential enrichment of antibodies able to rapidly recruit neutrophil activation, degranulation and cytokine release, but not other Fc-effector functions, in individuals who experienced severe disease compared with adults with mild disease or children who experience largely asymptomatic infection^66^. In addition, the FcγR3B upregulation has been linked to severe COVID-19 and might be used as a biomarker or therapeutic target^67^. Similarly, previous data have pointed to a critical role for neutrophils in natural protection against severe disease and death, where neutropenia was among the strongest risk factors for severe disease in patients with various hematological malignancies^67–69^. Likewise, in the present study we observed that the presence of FcγR3B-binding antibodies was enriched in individuals who were protected against COVID-19 breakthrough, linked to neutrophil-mediated immune complex clearance in the absence of neutrophil activation and inflammatory cytokine release. These data suggest that FcγR3B^+^ binding may temper the release of inflammatory cytokines that may drive early immune activation and cellular recruitment, potentially contributing to the initiation of the cytokine storm associated with the symptoms of COVID-19 (ref. ^70^).

Binding to FcγR3B, a GPI-anchor protein that does not lead to cellular activation^71^, resulted in comparable phagocytosis, in the absence of cytokine production, compared with antibodies unable to bind FcγR3B. These data suggest that FcγR3B may lead to rapid and robust viral clearance without inflammation. This muted immune-complex-mediated activation may be attributable to the fact that FcγR3B cannot signal. Instead, this receptor probably collaborates with other FcRs to help capture and clear antibody-opsonized material and thus may aid in clearance in the absence of cellular activation and the initiation of inflammatory cascades^72^. Conversely, previous studies suggested that IgA-mediated Fcα-receptor binding resulted in inflammatory activation of neutrophils^73^. Thus, the immune system may exploit distinct antibody isotype–FcR interactions to regulate neutrophil activity. Together these data suggest that viral clearance in the setting of a muted inflammatory response may be key to preventing the symptoms of COVID-19, whereas the generation of an inflammatory response may result in inflammatory cascades that may lead to dysregulated cellular recruitment, activation and disease associated with acute COVID-19. Importantly, IgA, IgG3 and FcγR2A can all activate neutrophils and were detected in all vaccinees, suggesting that antibody properties were not enhancing, but rather that, in the absence of FcγR3B, these antibody signals did not prevent COVID-19.

The ability of IgG to bind to FcRs is regulated by IgG subclass selection and Fc glycosylation^57^. Individuals who resisted COVID-19 had similar IgG titers and did not exhibit enhanced expression of non-IgG1 subclasses, pointing to the potential for Fc-glycosylation differences controlling FcγR3B binding. Along these lines, it is known that fucose-deficient IgGs bind preferentially to FcγR3a (refs. ^74,75^), whereas high galactosylation appears to further increase FcγR3a binding^76,77^. Galactose and sialic acid content has been shown to alter FcγR2 binding and activity^78^. However, the precise Fc glycans involved in binding preferentially to FcγR3B are not known. Instead, FcγR3A and FcγR3B are almost identical in their extracellular domains, marked by only four amino acid residue differences located in the antibody-binding domains, with the most important at position 129 (Gly129 in FcγR3A and Asp129 in FcγR3B) (refs. ^32,79^). The amino acid difference at position 129 leads to the presence of an additional N-linked glycan on FcγR3B that probably alters FcR binding to IgG subpopulations. FcγR3A has five *N*-glycans and FcγR3B has six; however, it is not fully clear if all sites are fully occupied^80^. Fc-glycan profiling of antibodies with the ability to interact with FcγR3B from controls compared with antibodies unable to interact with FcγR3B from controls clearly highlighted differences in Fc glycosylation across these groups. Thus, in line with previous studies that have noted striking differences in Fc glycosylation and natural COVID-19 outcomes^81–84^, the data presented in the present study provide additional mechanistic insights on how vaccines may exploit posttranslational humoral immune programming to drive enhanced control and clearance of the virus.

Whether some vaccinees are predisposed to generate particular antibody functional profiles that can interact with FcγR3B more effectively or whether some vaccinees simply failed to generate FcγR3B-binding antibodies remains unclear. In the present study, we observed equivalent vaccine-induced antibody titers and neutralization, but diverging Fc-glycan profiles across the vaccinees, suggesting that the cases responded well to vaccination, but generated a distinct profile of antibodies. Emerging data point to a role for vaccination in shaping Fc glycosylation^85^ and FcR binding can be further tuned using distinct adjuvants^86^. In the present study, IgG1 glycopeptide analysis pointed to the importance of lower galactosylation, sialylation and fucosylation, as well as increased levels of bisecting GlcNAc, because the glycan profile enriched on S-specific antibodies can interact with FcγR3B. Due to sample limitations, resulting in very low recovery of all four IgG subclasses, as well as the inability to distinguish IgG3/IgG4 glycopeptides by standard MS^43^, we were able to capture only IgG1 Fc-domain-specific glycosylation. Although systems serological analysis points to enhanced IgG3 responses among individuals who ultimately developed COVID-19, future studies, in which larger amounts of samples were collected outside of a regulated phase 2b study, may identify associated unique IgG-subclass-specific, antibody glycan profiles that may shape IgG1 activity, the dominant antibody subclass in the blood and the lung^87^. As mentioned above, although fucosylation was known to shape binding to FcγR3A (ref. ^88^), reduced galactosylation and sialylation are typical profiles enriched in inflammatory responses^89^ and may represent antibodies that are poised for rapid neutrophil-mediated clearance. Thus, learning to control Fc glycosylation via next-generation vaccine strategies may represent a unique opportunity to shape Fc-effector function to any pathogen. Moreover, specifically understanding how to induce FcγR3B-binding antibodies that can drive less inflammatory opsonophagocytosis may represent a unique approach to promoting viral clearance in the absence of inflammation and disease.

There are some limitations to our study. First, both natural infection and vaccination have been shown to drive distinct IgG Fc profiles^90^. Thus, specific vaccine platforms (for example, adenoviruses, messenger RNA, adjuvanted nanoparticles) are likely to drive distinct Fc subclass/isotype/glycosylation. Whether additional vaccine platforms are able to tune FcR-binding profiles and whether Fc-binding profiles decay differentially across platforms remain incompletely understood, but could provide key insights to guide boosting strategies. Second, it remains unclear whether these same biomarkers will track with protection across additional ChAdOx1 nCoV-19 trials globally, the same biomarkers will predict differential breakthrough across distinct vaccine platforms or the same biomarkers will predict vulnerability across newly emerging VOCs. Third, the cases and controls were matched based on standard demographic characteristics, such as sex (males versus females), age (20–60 years old), BMI categories (normal, obese, overweight and underweight) and race (black, mixed, others and white). Yet, Fc-glycan profiles also shift with HIV infection^89^, and are probably shaped by additional comorbidities (nutrition, coinfections, diabetes and so on). Thus, understanding additional differences across the populations may provide granular clues for the future posttranslational control of IgG glycosylation. Fourth, breakthrough infections can be caused by more frequent or greater exposures to SARS-CoV-2, which are difficult to account for in the setting of a phase 2b/3 trial, but could provide further insights into the mechanisms by which antibodies provide protection at different forces of infection. Therefore, future studies that can capture more information on risk of exposure and additional lifestyle factors could help refine the identification of mechanistic correlates of immunity, which could be used to improve vaccine design. Fifth, cytokine release by neutrophils was measured in the absence of complement (due to heat inactivation). Thus, although we do not think that complement affects the interpretation of our data, future studies examining the interaction of simultaneous FcR binding and complement activation would be of great interest. Sixth, data presented in the manuscript do not include the validation sample sets. Due to sample limitation (as a part of a phase 2b/3 trial), most experiments were performed as technical replicates. However, the functional validation by biochemical and molecular assays was implemented to further understand and confirm the unique signatures observed in the initial screening.

Given the importance of neutrophils as first responders in the setting of several respiratory infections^91,92^, the significance of the data presented in the present study points to a potentially important role for vaccine-induced, antibody-mediated innate immune activation as a key predictor of rapid viral clearance in the absence of inflammation as a surrogate of protection against COVID-19. Further evaluation of the biomarkers identified in the present study, as well as deeper evaluation of innate immune changes across populations (elderly and immunocompromised individuals and so on), may further uncover mechanisms that may be key to protection against COVID-19, and may help improve and optimize vaccine platforms that are ultimately aimed at inducing robust and durable protection against current and emerging VOCs.

## Methods

### Cohort description

Samples were collected from the multi-site, double-blind, randomized, placebo-controlled trial coordinated in South Africa at WITS-VIDA, aimed at assessing the safety and efficacy of two standard doses of the ChAdOx1 nCoV-19 vaccine^26^. The ChAdOx1 nCoV-19 vaccine was developed at the University of Oxford and WITS-VIDA was responsible for the conduct and oversight of the trial. Eligible participants were aged 18–65 years with no or well-controlled chronic medical conditions. The main exclusion criteria were HIV positivity at screening (for the efficacy cohort), previous or current laboratory-confirmed COVID-19, a history of anaphylaxis in relation to vaccination and morbid obesity (BMI ≥ 40). The vaccine was administered 21–35 days apart and compared with a saline (0.9% sodium chloride) placebo. A nucleic acid amplification test was used to detect SARS-CoV-2 infection at routine scheduled visits and whenever participants had any symptoms suggestive of COVID-19. Cases were selected as individuals who received two doses of the ChAdOx1 nCoV-19 vaccine and had confirmed COVID-19 more than 14 days post-boost by the positive PCR test result. Controls were randomly selected from a subset of seronegative participants at baseline, based on SARS-CoV-2 nucleocapsid-specific antibody testing and allocated to the vaccination arm, and were demographically matched based on sex (males versus females), age (20–60 years), BMI categories (normal, obese, overweight and underweight) and race (black, mixed, others and white) (Table 1).

The trial (Clinicaltrials.gov no. NCT04444674; Pan African Clinical Trials Registry no. PACTR202006922165132) was reviewed and approved by the South African Health Products Regulatory Authority and by the ethics committees of the University of the Witwatersrand, Cape Town, Stellenbosch and OxTREC before trial initiation. All participants were fully informed about the procedures and the possible risks, and all signed written informed consent documents.

### Antigens

Antigens used for Luminex-based assays are: SARS-CoV-2 D614G WT S (kindly provided by E. Saphire, La Jolla Institute for Immunology), SARS-CoV-2 S1 (Sino Biological), SARS-CoV-2 S2 (Sino Biological) and SARS-CoV-2 RBD (kindly provided by A. Schmidt, Ragon Institute) as well as SARS-CoV-2 VOCs, such as alpha B.1.1.7 S (LakePharma), beta B.1.351S (LakePharma), gamma P1 S (LakePharma), kappa B.1.617.1 S (Sino Biological) and delta B.1.617.2S (kindly provided by E. Saphire) and alpha B.1.1.7, beta B.1.351, gamma P1, kappa B.1.617.2 and delta B.1.617.2 RBDs (kindly provided by F. Krammer, Icahn School of Medicine at Mount Sinai) and hCoV-OC43 S (Sino Biological) and hCoV-HKU1 S (Immune Tech).

### Luminex profiling

Antibody isotyping (IgG1, IgG3, IgM and IgA) and Fcγ-receptor (Fcγ2A, Fcγ2B, Fcγ3A and Fcγ3B) binding were conducted using a customized multiplexed Luminex assay, and have been established under good clinical laboratory practice-like conditions^89,90^, including extensive testing to evaluate the number of washes required to avoid plasma-derived activation of innate immune cells^89,90^. Briefly, SARS-CoV-2 antigens were used to profile specific humoral immune responses. Antigens were coupled to magnetic Luminex beads (Luminex Corp) by carbodiimide–succinimidyl ester (NHS ester) coupling (Thermo Fisher Scientific). Antigen-saturated microspheres were washed and incubated with plasma samples at appropriate sample dilution (1:100 for IgG3, IgA and IgM; 1:500 for IgG1; 1:1,000 for all low-affinity Fcγ-receptors) for 2 h at 37 °C in 384-well plates (Greiner Bio-One). The high-affinity FcR was not tested due to its minimal role in tuning antibody effector function^32^. Unbound antibodies were washed away and antigen-bound antibodies were detected using a phycoerythrin (PE)-coupled detection antibody for each subclass and isotype (IgG1, IgG3, IgA1 and IgM; Southern Biotech) and Fcγ-receptors were fluorescently labeled with PE before addition to immune complexes (Fcγ2A, Fcγ2B, Fcγ3A and Fcγ3B; Duke Protein Production facility). After 1 h of incubation, plates were washed and flow cytometry was performed with an IQue (Intellicyt), and analysis was performed on Intellicyt ForeCyt (v.8.1). PE MFI is reported as a readout for antigen-specific antibody titers.

### Pseudovirus neutralization assay

The pseudovirus neutralization assay was conducted as previously described^26^. Briefly, neutralizing antibody activity was measured by assessing the inhibition of luciferase activity in HEK293 target cells expressing the angiotensin-converting enzyme 2 receptor, after preincubation of the pseudovirions with serial dilutions of the serum specimen. The expression of luciferase activity in target cells was inhibited in the presence of anti-SARS-CoV-2-neutralizing antibody. Titers were reported as the reciprocal of the serum dilution conferring 50% inhibition (ID_50_) of pseudovirus infection. To ensure that the measured neutralizing antibody activity is SARS-CoV-2-neutralizing antibody specific, each test specimen was assessed using a nonspecific pseudovirus (specificity control) that expresses a nonreactive envelope protein of one or more unrelated viruses (for example, avian influenza virus). Method validation included accuracy, repeatability, intermediate precision and linearity.

### Selection of samples for functional assays

Two pools of samples were selected based on their characteristic of interaction with FcγR2B or FcγR3B (pools of *n* = 5). The first pool contains samples from vaccinated COVID-19^+^ individuals who showed high binding to FcγR2B (>10^4^) and a lack of interactions with FcγR3B (<10^4^) (COVID-19^+^FcR2B^+^3B^−^ pool) based on Luminex data. The second pool contains samples from vaccinated COVID-19^−^ patients whose antibody profile revealed robust binding to FcγR3B (>10^4^) but lack of interaction with FcγR2B (<10^4^) (COVID-19^−^FcR2B^−^3B^+^ pool).

### Functional assays

ADCP, ADNP and ADCD assays were performed with heat-inactivated samples as previously described^91–93^. Due to the low availability of sample, pools of samples, representing the top extreme five FcR-binding profiles, were generated and run as technical replicates.

SARS-CoV-2 WT S protein was coupled to yellow–green (505/515-nm) or red–orange (565/580-nm) fluorescent NeutrAvidin-conjugated beads (Thermo Fisher Scientific) for ADCP/ADNP and ADCD, respectively. Immune complexes were formed by incubating the diluted pooled samples (ADCP and ADNP, 1:100 dilution) with the antigen-coupled beads for 2 h at 37 °C. For ADCP, 1.25 × 10^5^ THP-1 cells ml^−1^ were added to the immune complexes and incubated for approximately 18 h at 37 °C. After the incubation, THP-1 cells were washed and fixed with 4% paraformaldehyde (PFA; Alfa Aesar). For ADNP, the immune complexes were incubated with 5 × 10^5^ cells ml^−1^ of red blood cell (RBC)-lysed whole blood for 1 h at 37 °C. After incubation, cells were washed and stained for CD66b^+^ (BioLegend) to identify neutrophils and then fixed in 4% PFA.

For ADCD, the antigen-coupled beads were incubated with the diluted pooled samples (1:10 dilution) for 2 h at 37 °C to form immune complexes. The immune complexes were washed and lyophilized guinea-pig complement (Cedarlane), in gelatin veronal buffer with calcium and magnesium (GBV^++^) (Boston BioProducts), was added for 30 min (complement was reconstituted according to the manufacturer’s instructions). The deposition of complement was detected by a fluorescein-conjugated goat IgG fraction to guinea-pig complement C3 (Mpbio).

All the assays were acquired by flow cytometry with iQue and the analysis was performed using Intellicyt ForeCyt. The phagocytosis score was calculated (percentage cells positive × MFI of positive cells) for ADCP and ADNP. ADCD was reported as the median of C3 deposition.

### Cytokine release assay

The yellow–green (505/515-nm) fluorescent beads were coupled with SARS-CoV-2 WT S protein and incubated for 2 h at 37 °C with the diluted pooled samples (1:100 dilution) to form immune complexes. Once the incubation was completed, 5 × 10^5^ cells ml^−1^ of RBC-lysed whole blood were added to the immune complexes and incubated for 18 h at 37 °C. Subsequently, the supernatant was collected and ProcartaPlex Multiplex Immunoassay (Thermo Fisher Scientific) was performed according to the manufacturer’s instructions. Briefly, the supernatant was coupled with the cortisol beads, incubated with detection antibody and streptavidin–PE (Thermo Fisher Scientific). The data were acquired on Luminex FLEXMAP 3D (Thermo Fisher Scientific) and analyzed via xPONENT.

### Sample preparation for IgG Fc-glycosylation analysis

Anti-S IgG was captured using a set-up that resembles a conventional ELISA: IgGs were affinity captured from plasma using recombinant trimerized S protein-coated Maxisorp NUNC-Immuno plate (Thermo Fisher Scientific), whereas the total IgG was affinity captured using protein G Sepharose Fast Flow 4 beads, as described previously^94,95^. Antibodies were eluted using 100 mM formic acid and the samples were dried by vacuum centrifugation. Samples were reconstituted in 25 mM ammonium bicarbonate and subjected to tryptic cleavage. Three Visucon-F plasma standards (dating pre-COVID-19) and two blanks were included.

### IgG Fc-glycosylation analysis

Glycopeptides were separated and detected using an Ultimate 3000 high-performance liquid chromatography system (Thermo Fisher Scientific) linked to an Impact quadrupole time-of-flight mass spectrometer (Bruker Daltonics), as described^95^. Using this method, IgG1 glycoforms were assigned based on accurate mass and specific migration position in liquid chromatography (LC), excluding the possible glycopeptide-level interference of IgG3 with IgG2 and IgG4 (ref. ^94^).

### LC–MS data processing

MzXML files were generated from raw LC–MS spectra. An in-house-developed software, LaCyTools, was used for the alignment and targeted extraction of raw data^96^. Alignment was performed based on the average retention time of a minimum of three abundant IgG1 glycoforms. The targeted extraction list included analytes of the 2^+^ and 3^+^ charge states and was based on manual annotation of the mass spectra as well as on the literature^97,98^. A pre-COVID-19 plasma pool (Visucon-F) was measured in triplicate to assess method repeatability and used as a negative control. Signals were integrated by covering a minimum of 95% of the area of the isotopic envelope of glycopeptide peaks. The inclusion of an analyte for the final data analysis was based on quality criteria such as signal:noise ratio (>9), isotopic pattern quality (<25% deviation from the theoretical isotopic pattern) and mass error (within a ±20-p.p.m. range). Furthermore, analytes that were present in at least one out of four anti-S IgG1 spectra (25%) were included in the final analysis.

### Statistics

Data analysis was performed using R v.4.0.2 (2020-06-22). Comparisons between SARS-CoV-2-infected and -uninfected individuals were performed using a Mann–Whitney *U*-test test followed by a Benjamini–Hochberg correction. Antigen responses (for example, WT to beta) were compared using Wilcoxon’s signed-rank test followed by a Benjamini–Hochberg correction.

Classification models were trained to discriminate between infected and uninfected individuals using all the measured antibody responses. Before analysis, all data were normalized using *z*-scoring. Models were built using a combination of LASSO for feature selection and then classification using PLS-DA with the LASSO-selected features^99^ and R package ‘ropls’ v.1.20.0 (ref. ^100^) and ‘glmnet’ v.4.0.2. Model accuracy was assessed using tenfold crossvalidation. For each test-fold, LASSO-based feature selection was performed on logistic regression using the training set for that fold. LASSO was repeated 100× and features selected as at least 90× out of 100 were identified as selected features. A PLS-DA classifier was applied to the training set using the selected features and prediction accuracy was recorded. Selected features were ordered according to their variable importance in projection (VIP) score and the first two latent variables of the PLS-DA model were used to visualize the samples.

A cocorrelate network analysis was carried out to identify features that highly correlate with the LASSO-selected features and thus are potentially equally important for discriminating infected from uninfected individuals. Correlations for the cocorrelate network were performed using Spearman’s method followed by Benjamini–Hochberg multiple corrections^101^. The cocorrelate network was generated using R package ‘network’ v.1.16.0 (ref. ^102^). All other figures were generated using ggplot2 (ref. ^103^).

To assess the potential confounding effects of the demographics, for each feature null and full linear models were fit. Null models consisted of sex, age, BMI, race, alcohol and smoking status. The full models additionally included the group effect (COVID-19 uninfected or with COVID-19). A likelihood ratio test was then applied using the null and full models to obtain the significance of the group effect as *P* values. These *P* values were then plotted against the coefficient of the group effect.

### Reporting summary

Further information on research design is available in the Nature Portfolio Reporting Summary linked to this article.

## Online content

Any methods, additional references, Nature Portfolio reporting summaries, source data, extended data, supplementary information, acknowledgements, peer review information; details of author contributions and competing interests; and statements of data and code availability are available at 10.1038/s41590-023-01513-1.

## Supplementary information

Supplementary Information Supplementary Figs. 1–5.

Reporting Summary

## Data Availability

All anonymized data collected during the trial and associated with the present study can be provided. Requests should be directed to shabir.madhi@wits.ac.za or galit.alter@gmail.com. Source data are provided with this paper.

## Source data

Source Data Fig. 1 Raw data.
Source Data Fig. 2 Raw data.
Source Data Fig. 3 Raw data.
Source Data Fig. 4 Raw data.
Source Data Fig. 5 Raw data.
Source Data Fig. 6 Raw data.

## Code availability

Codes and scripts used for the present study have been deposited and can be found at GitHub: https://github.com/denizcizmeci/ChAdOx1_nCoV19.
