# Supplementary material for: ChAdOx1 nCoV-19 (AZD1222) vaccine-induced Fc receptor binding tracks with differential susceptibility to COVID-19
Source: Nat Immunol. 2023 Jun 15;24(7):1161–72. doi: 10.1038/s41590-023-01513-1 (PMC10307634; doi:10.1038/s41590-023-01513-1)
Supplement: Supplementary file 1 — Supplementary Figs. 1–5. [file 41590_2023_1513_MOESM1_ESM.pdf]

nature immunology

Article

https://doi.org/10.1038/s41590-023-01513-1

ChAdOx1 nCoV-19 (AZD1222) vaccine-induced Fc receptor binding tracks with differential susceptibility to COVID-19

In the format provided by the authors and unedited

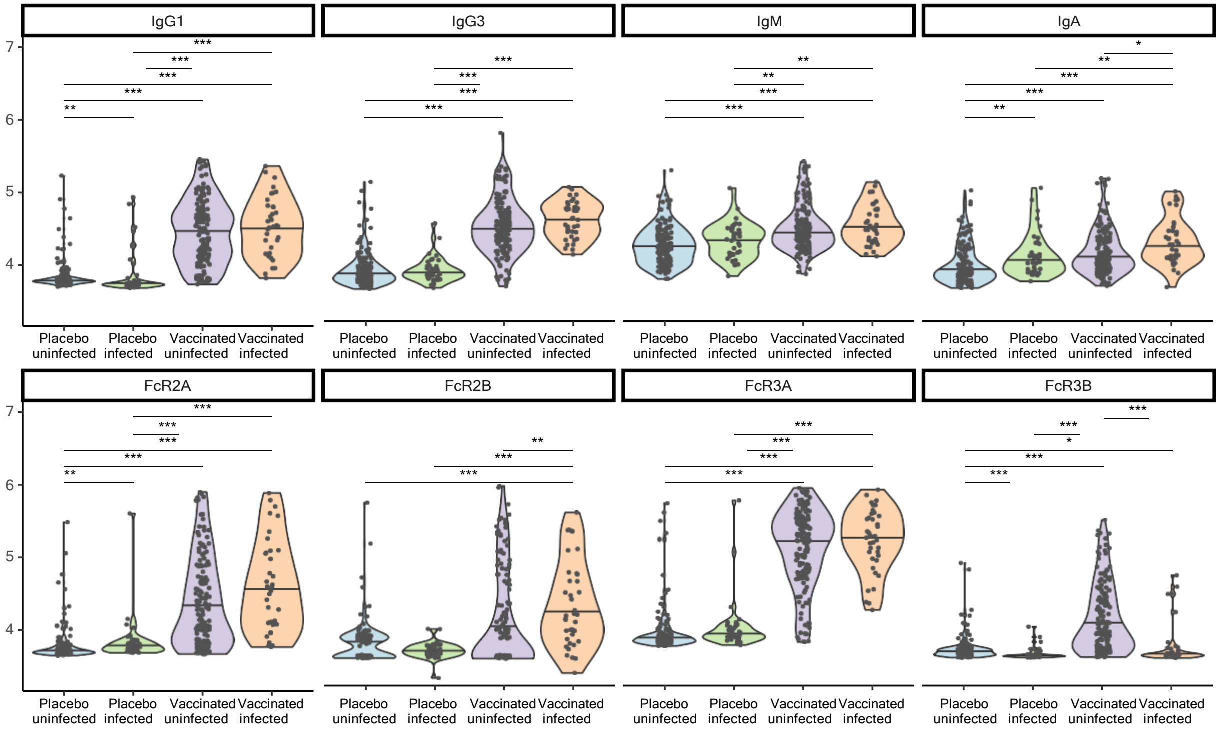

Supplemental Figure 1. S-specific antibody response and Fcγ-receptor binding profiles across ChAdOx1-nCoV-19 vaccinated and non-vaccinated groups. The violin plots show the univariate comparisons of WT Spike-specific and Fcγ-receptor binding profiles at peak immunogenicity (two weeks after the second injection) across ChAdOx1-nCoV-19 vaccinated and non-vaccinated (placebo) subjects that resisted COVID-19 (blue ($n = 140$) and violet ($n = 120$)) or that developed COVID-19 (green ($n = 30$) and orange ($n = 24$)) over the study period. A Mann-Whitney U-test was used to define statistical differences, and all values were corrected for multiple comparisons using the Benjamini-Hochberg (BH) method, with $p <$ 0.001 ***, $p < 0.01$ **, $p < 0.05$.

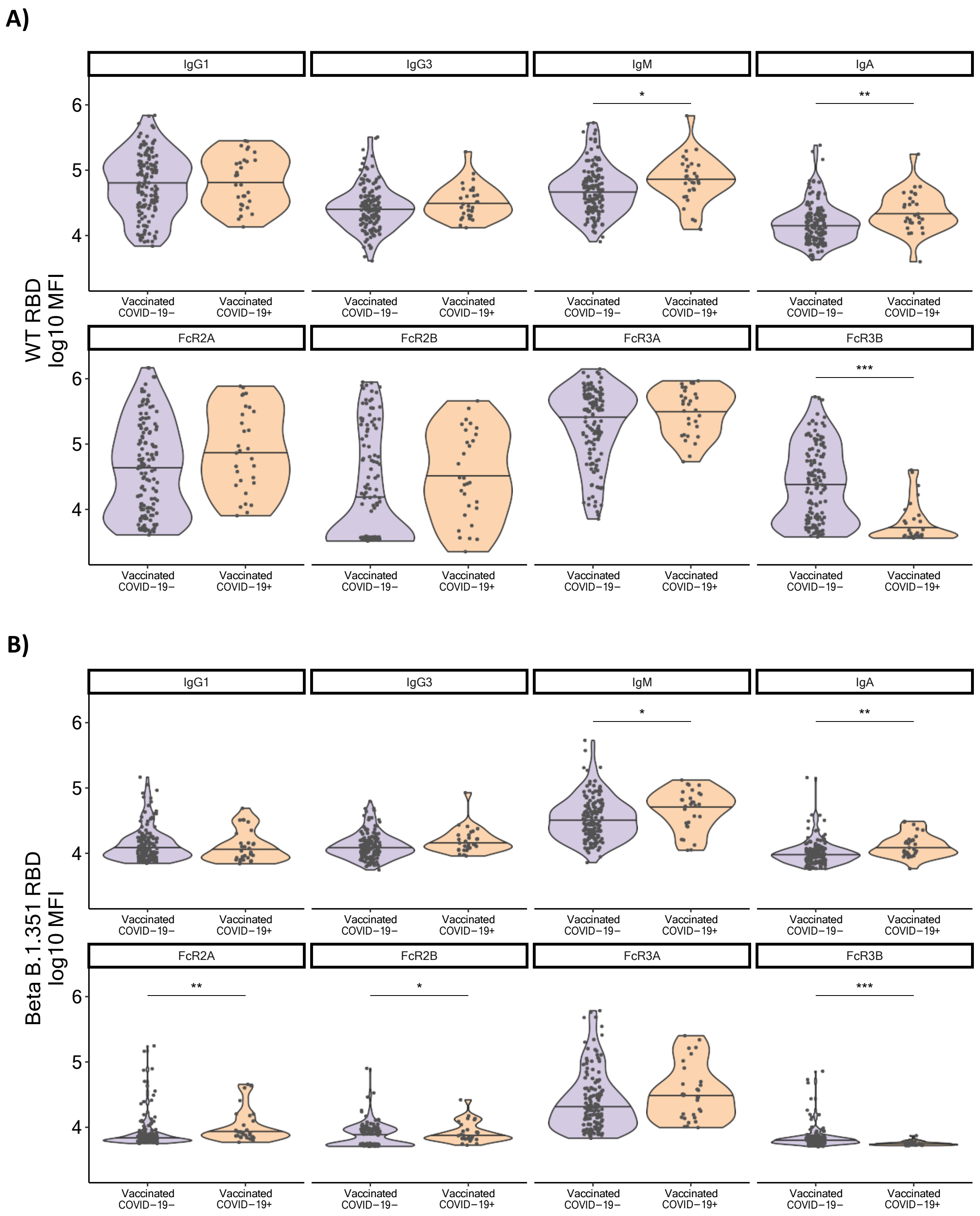

Supplemental Figure 2. RBD-specific antibody Fc-profiles across groups. The violin plots show the univariate comparisons of WT **(A)** and Beta **(B)** RBD-specific antibody titers and Fcγ-receptor binding profiles across ChAdOx1-nCoV-19 vaccinated subjects that resisted COVID-19 (violet, n = 140) or that developed COVID-19 (orange, n = 30) over the study period. A Mann-Whitney U-test was used to define statistical differences, and all values were corrected for multiple comparisons using the Benjamini-Hochberg (BH) method, with $p < 0.001$ ***, $p < 0.01$ **, $p < 0.05$ *.

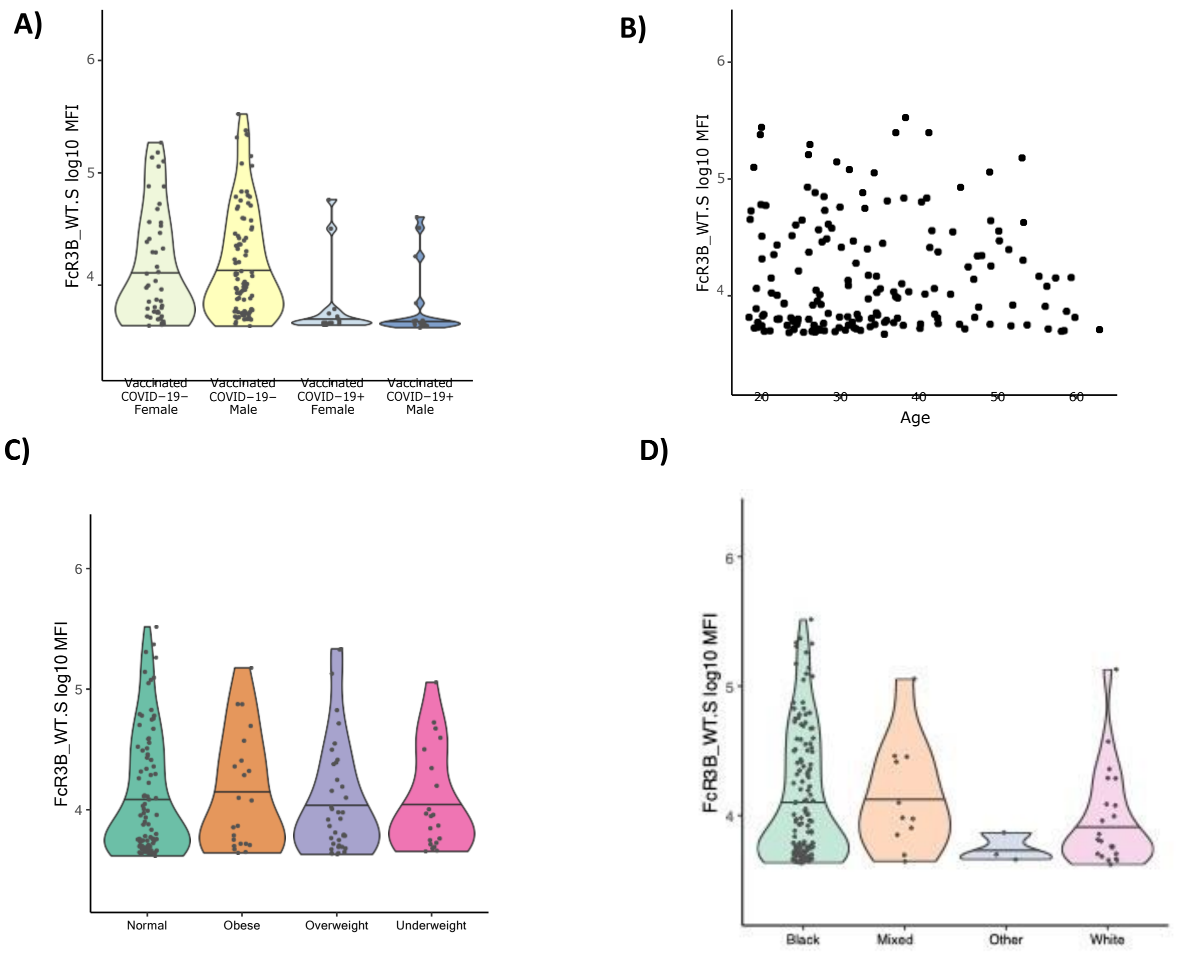

Supplemental Figure 3. A linear model adjusted for demographic confounders. Univariate graphs show FcγR3B levels based on **(A)** sex (males (*n = 108*) vs. females (*n = 62*), **(B)** age (20 to 60 years old), **(C)** BMI categories (normal (*n = 21)*, obese (*n = 22*), overweight (*n = 38*), and underweight (*n = 21*)), and **(D)** race (black (*n = 134*), mixed (*n = 11*), others (*n = 3*) and white (*n = 22*).

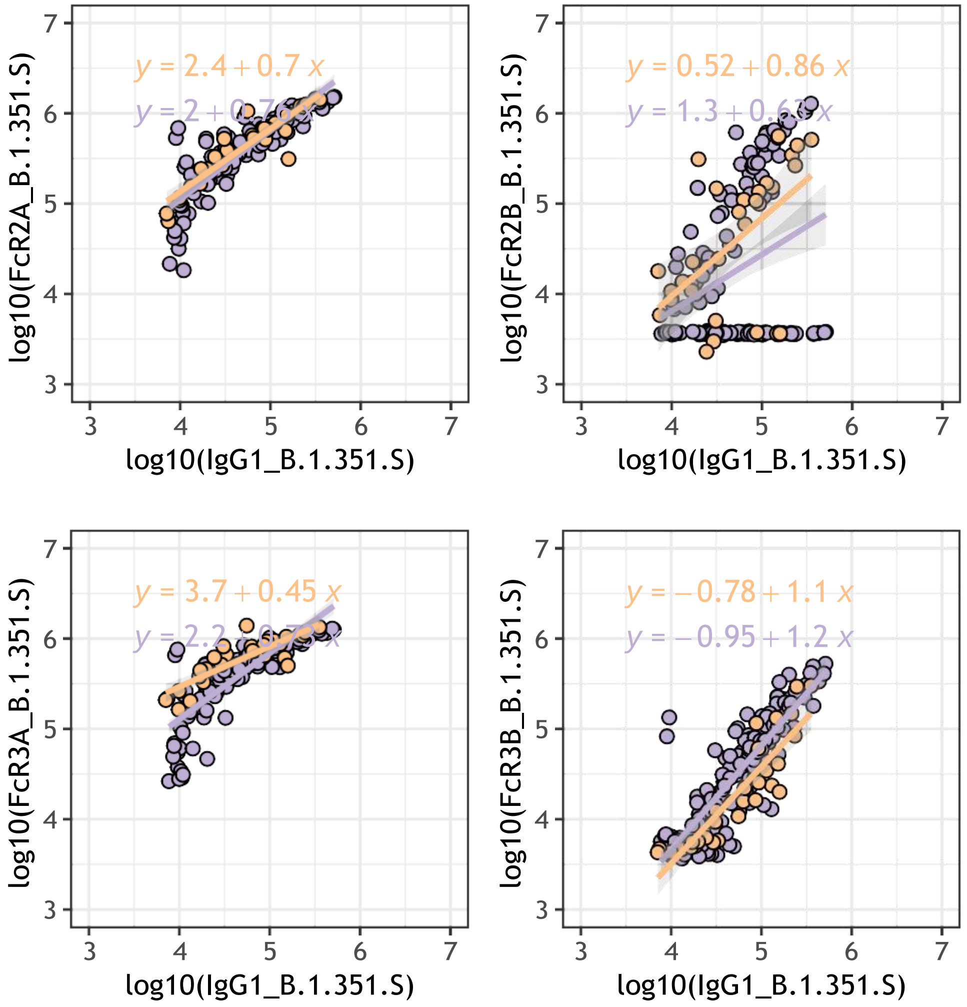

Supplemental Figure 4. The relationship between IgG1 titers and FcγR binding levels (FcγR2A, FcγR2B, FcγR3A, and FcγR3B) in the vaccinees that resisted COVID-19 or developed disease. Each dot represents single ChAdOx1-nCoV-19 vaccinated subjects that resisted COVID-19 (violet, *n = 140*) or that developed COVID-19 (orange, *n = 30*). The spearman correlations test was used to calculate the statistical significance.

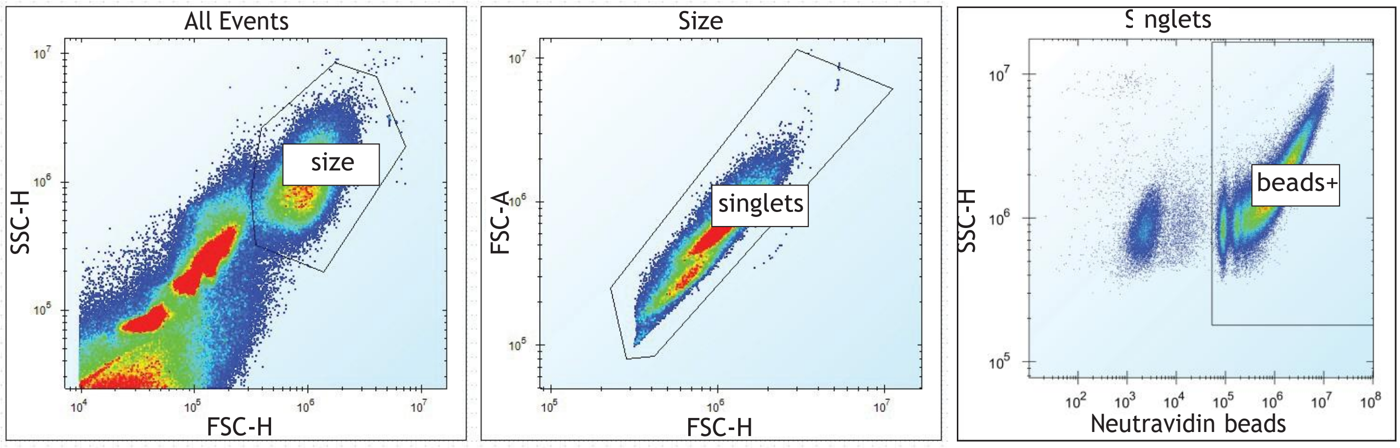

Supplemental Figure 5. Gating strategy for Luminex assay. Gating strategy and interpretation for Luminex assay. First, beads are defined by plotting side scatter height (SSC-H) versus forward scatter high (FSC-H). Doublet signals are excluded by plotting forward scatter area (FSC-A) versus forward scatter height (FSC-H). Positive beads are defined as positive for PE-conjugated and high side scatter (SSC-H).
