# Supplementary material for: ChAdOx1 nCoV-19 (AZD1222) vaccine-induced Fc receptor binding tracks with differential susceptibility to COVID-19
Source: Nat Immunol. 2023 Jun 15;24(7):1161–72. doi: 10.1038/s41590-023-01513-1 (PMC10307634; doi:10.1038/s41590-023-01513-1)
Supplement: Supplementary file 2 — Reporting Summary [file 41590_2023_1513_MOESM2_ESM.pdf]

nature research

Corresponding author(s): Galit Alter

Last updated by author(s): March 29th, 2023

Reporting Summary

Nature Research wishes to improve the reproducibility of the work that we publish. This form provides structure for consistency and transparency in reporting. For further information on Nature Research policies, see our Editorial Policies and the Editorial Policy Checklist.

Statistics

For all statistical analyses, confirm that the following items are present in the figure legend, table legend, main text, or Methods section.

n/a	Confirmed	
☐	☒	The exact sample size (*n*) for each experimental group/condition, given as a discrete number and unit of measurement
☐	☒	A statement on whether measurements were taken from distinct samples or whether the same sample was measured repeatedly
☐	☒	The statistical test(s) used AND whether they are one- or two-sided<br>*Only common tests should be described solely by name; describe more complex techniques in the Methods section.*
☐	☒	A description of all covariates tested
☐	☒	A description of any assumptions or corrections, such as tests of normality and adjustment for multiple comparisons
☐	☒	A full description of the statistical parameters including central tendency (e.g. means) or other basic estimates (e.g. regression coefficient) AND variation (e.g. standard deviation) or associated estimates of uncertainty (e.g. confidence intervals)
☐	☒	For null hypothesis testing, the test statistic (e.g. *F*, *t*, *r*) with confidence intervals, effect sizes, degrees of freedom and *P* value noted<br>*Give P values as exact values whenever suitable.*
☒	☐	For Bayesian analysis, information on the choice of priors and Markov chain Monte Carlo settings
☒	☐	For hierarchical and complex designs, identification of the appropriate level for tests and full reporting of outcomes
☒	☐	Estimates of effect sizes (e.g. Cohen's *d*, Pearson's *r*), indicating how they were calculated

Our web collection on statistics for biologists contains articles on many of the points above.

Software and code

Policy information about availability of computer code

Data collection	ForeCyt® Standard Edition 8.1 was used to collect Luminex, ADNP, ADCP and ADCD assay.<br>Cytokine release assay was run on LuminexTM FLEXMAP 3DTM (ThermoFisher) and analyzed via xPONENT<br>Ultimate 3000 high-performance liquid chromatography (HPLC) system (Thermo Fisher Scientific, Waltham, MA) hyphenated to an Impact quadrupole time-of-flight mass spectrometer (Bruker Daltonics, Billerica, MA)

Data analysis

Microsoft Excel 365 was used to compile experimental data and patient information.
Data analysis was performed using R version 4.0.2 (2020-06-22).
Classification models were trained to discriminate between infected and uninfected individuals using all the measured antibody responses. Prior to analysis, all data were normalized using z-scoring. Models were built using a combination of the least absolute shrinkage and selection operator (LASSO) for feature selection and then classification using partial least square discriminant analysis (PLS-DA) with the LASSO-selected features 101 using R package “ropls” version 1.20.0 (Thévenot et al., 2015) and “glmnet” version 4.0.2. Model accuracy was assessed using ten-fold cross-validation. For each test fold, LASSO-based feature selection was performed on logistic regression using the training set for that fold. LASSO was repeated 100 times, features selected at least 90 times out of 100 were identified as selected features. PLS-DA classifier was applied to the training set using the selected features, and prediction accuracy was recorded. Selected features were ordered according to their Variable Importance in Projection (VIP) score, and the first two latent variables (LVs) of the PLS-DA model were used to visualize the samples.
A co-correlate network analysis was carried out to identify features that highly correlate with the LASSO selected features and thus are potentially equally important for discriminating infected individuals from the uninfected. Correlations for the co-correlate network were performed using Spearman method followed by Benjamini-Hochberg multiple correction 102. The co-correlate network was generated using R package “network” version 1.16.0 103. All other figures were generated using ggplot2 104 plotting package.
Antibody Fc-glycan profiles were plotted in GraphPad Prism Version 9.3.1
Codes and scripts used for this study have been deposited and can be found at GitHub https://github.com/denizcizmeci/ChAdOx1_nCoV19.

For manuscripts utilizing custom algorithms or software that are central to the research but not yet described in published literature, software must be made available to editors and reviewers. We strongly encourage code deposition in a community repository (e.g. GitHub). See the Nature Research guidelines for submitting code & software for further information.

Data

Policy information about availability of data

All manuscripts must include a data availability statement. This statement should provide the following information, where applicable:

- Accession codes, unique identifiers, or web links for publicly available datasets
- A list of figures that have associated raw data
- A description of any restrictions on data availability

All anonymized data collected during the trial and associated with this study can be provided. Request should be directed to shabir.madhi@wits.ac.za or galter@mgh.harvard.edu. Data requestors will need to sign a data access agreement to gain access, and access will be granted for non-commercial research purposes only.

Field-specific reporting

Please select the one below that is the best fit for your research. If you are not sure, read the appropriate sections before making your selection.

☒ Life sciences ☐ Behavioural & social sciences ☐ Ecological, evolutionary & environmental sciences

For a reference copy of the document with all sections, see nature.com/documents/nr-reporting-summary-flat.pdf

Life sciences study design

All studies must disclose on these points even when the disclosure is negative.

Sample size	No sample size calculation was performed. We used all samples received from the multi-site, double-blind, randomized, placebo-controlled trial, co-ordinated in South Africa at WITS-VIDA.
Data exclusions	The main exclusion criteria were human immunodeficiency virus (HIV) positivity at screening, previous or current laboratory-confirmed Covid-19, a history of anaphylaxis in relation to vaccination, and morbid obesity (body-mass index ≥40).
Replication	All experiments were run in duplicates and Luminex and functional assays (ADCD, ADNP and ADCD) repeated for most of the samples. Results between repeats were comparable.
Randomization	Samples were randomly distributed in 96 well plates
Blinding	Investigators were blinded during data collection. Group allocation had to be revealed at the end of analysis to perform groups comparisons.

Reporting for specific materials, systems and methods

We require information from authors about some types of materials, experimental systems and methods used in many studies. Here, indicate whether each material, system or method listed is relevant to your study. If you are not sure if a list item applies to your research, read the appropriate section before selecting a response.

Materials & experimental systems

n/a	Involved in the study
☐	☒ Antibodies
☐	☒ Eukaryotic cell lines
☒	☐ Palaeontology and archaeology
☒	☐ Animals and other organisms
☐	☒ Human research participants
☐	☒ Clinical data
☒	☐ Dual use research of concern

Methods

n/a	Involved in the study
☒	☐ ChIP-seq
☐	☒ Flow cytometry
☒	☐ MRI-based neuroimaging

Antibodies

Antibodies used

1 .Mouse Anti-Human IgG1-PE (Southern-Biotech, #9054-09, clone:HP6001)
2. Mouse Anti-Human IgG2-PE (Southern-Biotech, #9060-09, clone:31-7-4)
3. Mouse Anti-Human IgG3-PE (Southern-Biotech, #9210-09, clone:HP6050)
4. Mouse Anti-Human IgG4-PE (Southern-Biotech, #9200-09, clone:HP6025)
5. Mouse Anti-Human IgM-PE (Southern-Biotech, #9020-09, clone:SA-DA4)
6. Mouse Anti-Human IgA1-PE (Southern-Biotech, #9130-09, clone: B3506B4)
7. Anti-guinea pig complement C3 goat IgG fraction (MP Biomedical, #855385. polyclonal)
8. Anti-human CD66b Pacific Blue (Biolegend, #305112 ,clone G10F5)

Validation

All antibodies were previously validated. The use of antibodies 1-6 was previously validated and described in: Brown EP, Licht AF, Dugast AS, Choi I, Bailey-Kellogg C, Alter G, et al. High-throughput, multiplexed IgG subclassing of antigen-specific antibodies from clinical samples. J Immunol Methods. 2012;386(1-2):117-23.
Antibody 7 was validated and described here: Fischinger, S., J. K. Fallon, A. R. Michell, T. Broge, T. J. Suscovich, H. Streeck, and G. Alter. 2019. 'A high-throughput, bead-based, antigen-specific assay to assess the ability of antibodies to induce complement activation', J Immunol Methods, 473: 112630.
Antibody 8: Karsten, C. B., N. Mehta, S. A. Shin, T. J. Diefenbach, M. D. Slein, W. Karpinski, E. B. Irvine, T. Broge, T. J. Suscovich, and G. Alter. 2019. 'A versatile high-throughput assay to characterize antibody-mediated neutrophil phagocytosis', J Immunol Methods, 471: 46-56.
All antibodies are well established and quality controlled by the manufacturer. Additional information and references can be obtained on the company websites.

Eukaryotic cell lines

Policy information about cell lines

Cell line source(s)

THP-1 cells (ATCC #TIB-202), HEK (ATCC, #CRL-1573

Authentication

None of the cell lines used were authenticated.

Mycoplasma contamination

Cell lines were not tested for mycoplasma contamination

Commonly misidentified lines
(See ICLAC register)

No commonly misidentified lines were used.

Human research participants

Policy information about studies involving human research participants

Population characteristics

We analyzed vaccinees that developed Beta-variant COVID-19 (n = 30) and a demographically matched set of vaccinated controls who remained free of COVID-19 (n = 140) as well as the placebo group (n = 144). Population characteristic was described in Table 1. Briefly, the cohort include 108 male (63.5%) and 62 female (36.5%); median age 31 (from 18 to 62 years old); race of participants: 78.8% Black; 12.9% white, 6.4% mixed and 1.6% others.

Recruitment

Samples were collected from the multi-site, double-blind, randomized, placebo-controlled trial, co-ordinated in South Africa at WITS-VIDA, aimed at assessing the safety and efficacy of two standard doses of the ChAdOx1-nCoV-19 vaccine. The ChAdOx1-nCoV-19 vaccine was developed at the University of Oxford, and WITS-VIDA was responsible for the conduct and oversight of the trial.

Ethics oversight

The trial (Clinicaltrials.gov number: NCT04444674; Pan African Clinical Trials Registry number: PACTR202006922165132) was reviewed and approved by the South African Health Products Regulatory Authority and by the ethics committees of the University of the Witwatersrand, Cape Town, Stellenbosch, and OxTREC before trial initiation. All participants were fully informed about the procedures and the possible risks, and all signed written informed consent documents.

Note that full information on the approval of the study protocol must also be provided in the manuscript.

Clinical data

Policy information about clinical studies

All manuscripts should comply with the ICMJE guidelines for publication of clinical research and a completed CONSORT checklist must be included with all submissions.

Clinical trial registration	Clinicaltrials.gov number: NCT04444674; Pan African Clinical Trials Registry number: PACTR202006922165132
Study protocol	https://www.nejm.org/doi/suppl/10.1056/NEJMoa2102214/suppl_file/nejmoa2102214_protocol.pdf
Data collection	Data was collected during clinical trial in South Africa performed between June and November 2020. The trial (Clinicaltrials.gov number: NCT04444674; Pan African Clinical Trials Registry number: PACTR202006922165132) was reviewed and approved by the South African Health Products Regulatory Authority and by the ethics committees of the University of the Witwatersrand, Cape Town, Stellenbosch, and OxTREC before trial initiation. All participants were fully informed about the procedures and the possible risks, and all signed written informed consent documents. A nucleic acid amplification test was used to detect SARS-CoV-2 infection at routine scheduled visits and whenever participants had any symptoms suggestive of COVID-19. Cases were selected as individuals who received two doses of the ChAdOx1-nCoV-19 vaccine and had confirmed COVID-19 more than 14 days post-boost by the positive PCR test result. Controls were randomly selected from a subset of seronegative participants at baseline based on SARS-CoV-2 nucleocapsid-specific antibody testing and allocated to the vaccination arm and were demographically matched based on sex (males vs. females), age (20 to 60 years old), BMI categories (normal, obese, overweight, and underweight), and race (black, mixed, others and white).
Outcomes	Outcome of the study was described in original publication Madhi, S. A. et al. Efficacy of the ChAdOx1 nCoV-19 Covid-19 Vaccine against the B.1.351 Variant. N Engl J Med 384, 1885-1898, doi:10.1056/NEJMoa2102214 (2021). Briefly, phase 1b–2 trial in South Africa aimed to evaluate the safety, carcinogenicity, and efficacy of the ChAdOx1 nCoV-19 vaccine in preventing symptomatic Covid-19. The interim analysis iwas limited to address the primary objective, such as evaluating safety and the primary and key secondary objectives evaluating vaccine efficacy, including efficacy specifically against the B.1.351 variant. Furthermore, the trial reported immunogenicity of ChAdOx1 nCoV-19 and on post hoc pseudovirus and live-virus neutralization assay investigations of the sensitivity of the original D614G virus and the B.1.351 variant to vaccine-elicited antibodies.

Flow Cytometry

Plots

Confirm that:

☒ The axis labels state the marker and fluorochrome used (e.g. CD4-FITC).

☒ The axis scales are clearly visible. Include numbers along axes only for bottom left plot of group (a 'group' is an analysis of identical markers).

☒ All plots are contour plots with outliers or pseudocolor plots.

☒ A numerical value for number of cells or percentage (with statistics) is provided.

Methodology

Sample preparation	Immune complexes were formed by incubating the diluted pooled samples (ADCP and ADNP 1:100 dilution) with the antigen-coupled beads for 2 h at 37C. For ADCP, 1.25x105 THP-1 cells/mL were added to the immune complexes and incubated for approximately 18 h at 37C. After the incubation, THP-1 cells were washed and fixed with 4% paraformaldehyde (PFA) (Alfa Aesar). For ADNP, the immune complexe were incubated with 5 x105 cells/ml of RBC-lysed whole blood for 1 h at 37C. After incubation, cells were washed and stained for CD66b+ (Biolegend) to identify netrophiles, and then fixed in 4% PFA.<br>For ADCD, the antigen-coupled beads were incubated with the diluted pooled samples (1:10 dilution) for 2 h at 37C to form immune complexes. The immune complexes were washed and lyophilized guinea pig complement (Cedarlane) in gelatin veronal buffer with calcium and magnesium (GBV++) (Boston BioProducts) was added for 30 min (complement was reconstituted according to manufacturer's instruction). The deposition of complement was detected by fluorescein-conjugated goat IgG fraction to guinea pig Complement C3 (Mpbio).<br><br>Sample preparation for IgG Fc glycosylation analysis<br>Anti-S IgG was captured using a setup that resembles a conventional ELISA: IgGs were affinity-captured from plasma using recombinant trimerized S protein-coated Maxisorp NUNC-Immuno plate (Thermo Fisher Scientific, Roskilde, Denmark), whereas the total IgG was affinity-captured using protein G Sepharose Fast Flow 4 beads, as described previously 59,60. Antibodies were eluted using 100 mM formic acid and the samples were dried by vacuum centrifugation. Samples were reconstituted in 25 mM ammonium bicarbonate and subjected to tryptic cleavage. 3 Visucon-F plasma standards (dating pre-COVID-19) and two blanks were included
Instrument	IntelliCyt® iQue Screener PLUS; Ultimate 3000 high-performance liquid chromatography (HPLC) system (Thermo Fisher Scientific, Waltham, MA) hyphenated to an Impact quadrupole time-of-flight mass spectrometer (Bruker Daltonics, Billerica, MA
Software	ForeCyt® Standard Edition 8.1 was used to collect and analyze the data.<br>MzXML files were generated from raw liquid chromatograph – mass spectrometry (LC-MS) spectra. An in-house developed software, LaCyTools was used for the alignment and targeted extraction of raw data

Cell population abundance	Primary neutrophils were identified by CD66b surface expression. CD66b expression was donor dependent bu usually >95% within the single cell gate.<br>All single cells were considered THP-1 cells.
Gating strategy	All events were gated for granulocutes using FSC-H and SSC-H and single cells subsequently selected using SSC-A and SSC-H.<br>For ADNP assay: primary neutrophils selected for CD66b expression. Phagocytic cells identified in the BL4-H channel.<br>Phagocytic THP-1 cells were directly determined in the single cell population.

☒ Tick this box to confirm that a figure exemplifying the gating strategy is provided in the Supplementary Information.
